# Quantitative Nanomechanical Mapping of Polyolefin Elastomer at Nanoscale with Atomic Force Microscopy

**DOI:** 10.1186/s11671-021-03568-1

**Published:** 2021-07-03

**Authors:** Shuting Zhang, Yihui Weng, Chunhua Ma

**Affiliations:** Collaborative Innovation Center of Henan Province for Green Manufacturing of Fine Chemicals, Key Laboratory of Green Chemical Media and Reactions, Ministry of Education, School of Chemistry and Chemical Engineering, Henan Normal University, Xinxiang, 453007 Henan China

**Keywords:** Low density polyolefin, PeakForce quantitative nanomechanical mapping, Fast force volume, Young’s modulus, DMT model, Atomic force microscopy

## Abstract

Elastomeric nanostructures are normally expected to fulfill an explicit mechanical role and therefore their mechanical properties are pivotal to affect material performance. Their versatile applications demand a thorough understanding of the mechanical properties. In particular, the time dependent mechanical response of low-density polyolefin (LDPE) has not been fully elucidated. Here, utilizing state-of-the-art PeakForce quantitative nanomechanical mapping jointly with force volume and fast force volume, the elastic moduli of LDPE samples were assessed in a time-dependent fashion. Specifically, the acquisition frequency was discretely changed four orders of magnitude from 0.1 up to 2 k Hz. Force data were fitted with a linearized DMT contact mechanics model considering surface adhesion force. Increased Young’s modulus was discovered with increasing acquisition frequency. It was measured 11.7 ± 5.2 MPa at 0.1 Hz and increased to 89.6 ± 17.3 MPa at 2 kHz. Moreover, creep compliance experiment showed that instantaneous elastic modulus *E*_*1*_, delayed elastic modulus *E*_*2*_, viscosity *η*, retardation time *τ* were 22.3 ± 3.5 MPa, 43.3 ± 4.8 MPa, 38.7 ± 5.6 MPa s and 0.89 ± 0.22 s, respectively. The multiparametric, multifunctional local probing of mechanical measurement along with exceptional high spatial resolution imaging open new opportunities for quantitative nanomechanical mapping of soft polymers, and can potentially be extended to biological systems.

## Introduction

With the rapid progress of advanced polymerization techniques, it comes with growing interest in polymer morphologies and their mechanical assessment [1]. One popular class of polymers is elastomers. Elastomeric nanostructures are normally expected to fulfill an explicit mechanical role and therefore their mechanical properties are pivotal to affect material performance. They normally exhibit spatial and temporal heterogeneities in their properties. How their nanoscale structure and properties are linked to micro- counterparts that finally lead to bulk properties are not fully understood [2–8]. Their versatile applications demand a thorough understanding of the mechanical properties. Polyolefin elastomers (PE) has generated great interest in a number of research and industrial fields, such as high voltage cable [9], nanofiber membrane [10], reusable materials [11], and immiscible polymer systems [12]. It has proven to be an effective and reliable model polymer system for nanomechanical measurement [13, 14]. Despite its wide applications, the elastic modulus measurement of low density PE (LDPE) remains challenging for several reasons [15]. Firstly, they are viscoelastic, meaning their mechanical responses are time-dependent. Secondly, large surface forces complicate the indentation process. Thirdly, robust models that faithfully describe contact mechanics are scarce. Multiple studies have been conducted using indentation to measure the mechanical properties of LDPE. Noteworthy advances have been accomplished to understanding the modulus of LDPE. For examples, how temperature [16], linear low-density polyethylene [17], nano-powder mixture [18] affect its Young’s modulus have been reported. However, the predominant majority of these studies lack high spatial resolution and the results cannot satisfy the increasing interest in quantitative characterization at nanoscale. Many researchers have turned to alternative techniques, such as atomic force microscopy (AFM) based force measurements [1, 15].

Soon after its invention in 1980s, AFM has been established to be a powerful tool to interrogate samples’ mechanical properties. Historically, AFM is capable of keeping track of vertical deflection change when Z piezo position is ramped. The correspondent force load and unload trajectories are recorded (force–displacement curve). The force–displacement curve is then processed to force-distance curve, which is fitted with different contact mechanics models. It can be done either in a single location measurement (single force ramp) or in a matrix array fashion, so called force volume (FV). The application of the conventional force measurement is severely time-consuming owing to its slow sampling rate, which is intrinsically limited by the instrument. The slow acquisition rate has been improved by a newly coined method named fast force volume (FFV). It can be operated from 0.1 Hz up to about 200 Hz. The underlying working mechanism for FFV relies on the smoothening of the triangular drive signal at transition, leading to fast turnaround between approach and retract. Despite the unprecedented technical advances, there is still room for improvement in terms of force sampling rate. The PeakForce tapping (PFT) based quantitative nanomechanical mapping (PFQNM) is an emerging approach that leverages its high-resolution imaging capability and mapping mechanical properties concurrently. PFQNM is complimentary to regular force volume by bringing up sampling speed up to 2 kHz. Therefore, PFQNM, force volume jointly with fast force volume make up for four orders of magnitude in terms of force loading/unloading rate. The aforementioned approaches are instrumental in terms of measuring elastic modulus, e.g. Young’s modulus. However, they provide little or no dynamic mechanical behavior of sample. Thankfully, AFM offers another unique feature that is called creep compliance experiment [19]. In this design, the AFM probe is brought into contact with sample surface at a preload force. The probe is then held still with fixed applied force. While the stress is the constant, the material undergoes creep. The AFM monitors the indentation change as a function of time. The acquired data is then subject to model fit. A wealthy of information on the dynamic mechanical properties of materials can be extracted from such measurement. If all abovementioned techniques are assembled together, they are promising to effectively investigate time dependent mechanical properties for soft polymers.

In addition to force mapping, PFT is an exceptional tool [20] for topography imaging. In PFT, the Z piezo drives the whole probe holder up and down at low frequencies, normally in the range of 0.5k–2k Hz. It provides superior fine control of the force since it gives direct feedback on vertical deflection of a soft cantilever. The capability of successfully controlling the maximum interaction force earned its name as PeakForce tapping. In addition, it preserves high resolution as well as low invasiveness. These appealing characteristics make PFT an ideal technique in topography imaging of soft biological specimen and polymer samples. For instance, peak force tapping mode has been successfully applied to investigate the adhesion force between conducting polymers [21] and biorecognition event of single molecules [22]. To date, PFQNM has gained broad interest in characterizing the mechanical properties of a wide range of materials, including hardened cement paste [23], living cells [24], amyloid fibrils [25], polymer matrix composite [26–28] and a variety of polymers [29]. Since high resolution height image is also collected, it provides convenience to correlate local mechanical properties with sample topography at nanoscale.

In this study, the time-dependent modulus of a LDPE sample has been assessed utilizing a number of approaches. Specifically, the ramp frequency is changed discretely from 0.1 up to 2k Hz. Rigorous calibrations are done, and data are fitted with a proper Derjaguin–Muller–Toporov (DMT) contact mechanics model. Increasing Young’s modulus has been discovered with increasing ramp frequency. Creep compliance experiment was carried out to further understand the dynamic mechanical behavior of LDPE. Instantaneous elastic modulus *E*_1_, delayed elastic modulus *E*_2_, viscosity *η*, and retardation time *τ* has been extracted from the standard linear solid (SLS) model fit. The multiparametric mechanical measurement as well as unprecedented high spatial resolution topography imaging has been successfully exploited for quantitative nanomechanical mapping of soft polymers such as LDPE, and can potentially be extended to biological systems.

## Materials and Methods

### Materials

A PeakForce QNM sample kit was purchased from Bruker Co. (Santa Barbara, CA). A polymer blend sample, a sapphire sample and a tip check sample were included in the kit. The polymer blend sample is comprised of a thin film of polystyrene (PS) mixed with low density polyolefin (LDPE). The samples were mounted on metal pucks using double-sided tape and used as received. According to the manufacture, a blend of PS and LDPE (ethylene-octene copolymer) were spin-cast onto a silicon substrate, creating a film with varying material properties. RTESPA-150 probes were purchased from Bruker Co. (Santa Barbara, CA) with nominal spring constant of 5 N/m. The backside of probe cantilevers was coated with a thin aluminum layer to enhance laser deflection.

### Calibrations

A Dimension ICON AFM (Bruker Co., Santa Barbara, CA) equipped ScanAsyst mode was utilized to conduct calibrations and mechanical measurements. Calibrations on cantilever deflection sensitivity, cantilever spring constant and tip radius were carried out for force ramp and force volume. Three probes from the same batch were used in this study. The calibration protocols were as follows. Cantilever deflection sensitivity was calibrated by performing a force ramp through the so called touch calibration approach, in which a RTESPA-150 probe was brought onto a very hard surface, in this case the sapphire sample. The ramp output was selected for Z. Ramp size was kept at 200 nm and the relative trigger threshold was fixed at 0.3 V above the baseline background. After a force versus Z piezo displacement curve was collected, a pair of lines were used to define the most linear part of the contact region. The deflection sensitivity would be automatically calibrated and saved once clicking update deflection sensitivity. The measured deflection sensitivity was 44.7 ± 4.2 nm/V (*n* = 3). Next, thermal tune was performed to acquire the vibration spectrum of the cantilever in free air due to thermal energy. The resonance frequency peak was highlighted and fitted by the real time NanoScope software that was provided by the AFM manufacturer (Bruker Co. Santa Barbara, CA). Based on the theory of equipartition theorem,

1 $$\frac{1}{2}k_{{\text{B}}} T = \frac{1}{2}kd^{2}$$

where $k_{{\text{B}}}$ is the Boltzmann constant, $T$ is the absolute temperature in Kelvin, and $d$ is the root mean square value of the cantilever vibration amplitude. The spring constant $k$ was calculated accordingly by taking into account a correction factor of 1.09. Tip radius was estimated by cautiously scanning the probe across the tip check sample. The sample is comprised of titanium that has pointy ends at some regions. Each sharp end would capture a part of tip shape. In the end, the sample topography image could be used to reconstruct the tip shape, which was presumed to be a sphere. To precisely estimate the tip radius, an indentation depth was also needed. The indentation depth (18.3 ± 2.6 nm, *n* = 3) was obtained by measuring the distance between the zero separation and the lowest point in jump in contact. The effective tip radius was thereby calibrated by substituting the indentation value in the Height 1 from apex on the tip check image.

Sync Distance and PFT Amplitude Sensitivity are unique to PFQNM technique. They need to be calibrated as well. Sync distance is defined as a time constant at which the Z piezo reaches the lowest position. PFT Amplitude Sensitivity is referred to as a scaling factor that transfers the digitally input drive signal to the physically Z piezo displacement. Its accuracy ensures the Z piezo moves as desired. Both Sync Distance and PFT Amplitude Sensitivity were calibrated on the sapphire sample using touch calibration approach. Notably, the Sync Distance and PFT Amplitude Sensitivity are frequency dependent. Both were calibrated at discrete frequencies. In this work, a wide range of frequencies were selected spanning from 0.125k to 2k Hz.

### PFQNM Quantitative Nanomechanical Mapping

RTESPA-150 probes were loaded for quantitative nanomechanical mapping of the LDPE sample. The calibrated spring constants were 3.9 ± 1.4 N/m (n = 3). Upon scanning, user set the force setpoint at 5 nN while letting the ScanAsyst auto control to optimize the imaging acquiring rate (scan rate), feedback gain and Z range. The digital pixel was kept at 256 × 256 per image. The PFT frequency was varied from 2k to 0.125k Hz between experiments to produce time dependent force loading and unloading. For 100 nm PFT amplitude at 2 kHz PFT frequency, the corresponding force loading rate was 0.8 mm s^−1^. The Poisson’s ratio for viscoelastic LDPE was assumed to be 0.35 [13]. A 5 µm × 5 µm survey region was scanned simultaneously with topography and mechanical measurements. The NanoScope controller had sufficient bandwidth to compute mechanical data and display them in real time software channels. Those data were saved in raw images for further offline analysis. Therefore, a number of image channels were captured, including height sensor, DMT modulus, adhesion map, indentation and energy dissipation channels. Once the LDPE and polystyrene components were identified. High spatial resolution PFQNM measurements on LDPE were performed on a 0.5 µm × 0.5 µm scan.

### AFM Force Ramp and Fast Force Volume

Force ramp and fast force volume were achieved by ramping Z piezo displacement while monitoring vertical deflection of cantilever. The ramp size was 200 nm. Low trigger force setpoint at 5 nN was accomplished by a constant background subtraction mechanism that excludes the deflection drift during ramp process. A force ramp sampling array was defined over a 0.5 µm × 0.5 µm region. The ramp rates were 0.1 Hz, 1 Hz, 10 Hz, 20 Hz, 61 Hz and 122 Hz. For 1 Hz ramp rate and 200 nm ramp size, the corresponding force loading rate was 400 nm s^−1^. There were 16 × 16 ramp curves collected for 0.1 Hz and 1 Hz while 128 × 128 ramp curves for 10 Hz, 20 Hz, 61 Hz and 122 Hz.

### Creep Experiment

The Stargate scanner was drift calibrated for creep experiment. RTESPA-150 probes were brought into contact onto a neat LDPE region of the PS/LDPE sample until they reached a preset force load at 2 nN. The surface controls feature of NanoScope software enabled keeping the probe on the sample for certain time period, in this case 5 s. This period was named hold segment. The applied force was maintained constant by holding the trigger force. A thousand and twenty four data points were collected for the hold segment. Both height sensor versus time and deflection error (force) versus time were acquired. At least 50 creep curves were captured on randomly selected locations. Three independent experiments were conducted. A blank control experiment was carried out on the sapphire sample. As expected, no appreciable change in Z was observed.

### Experimental Setup

**Fig. 1:**
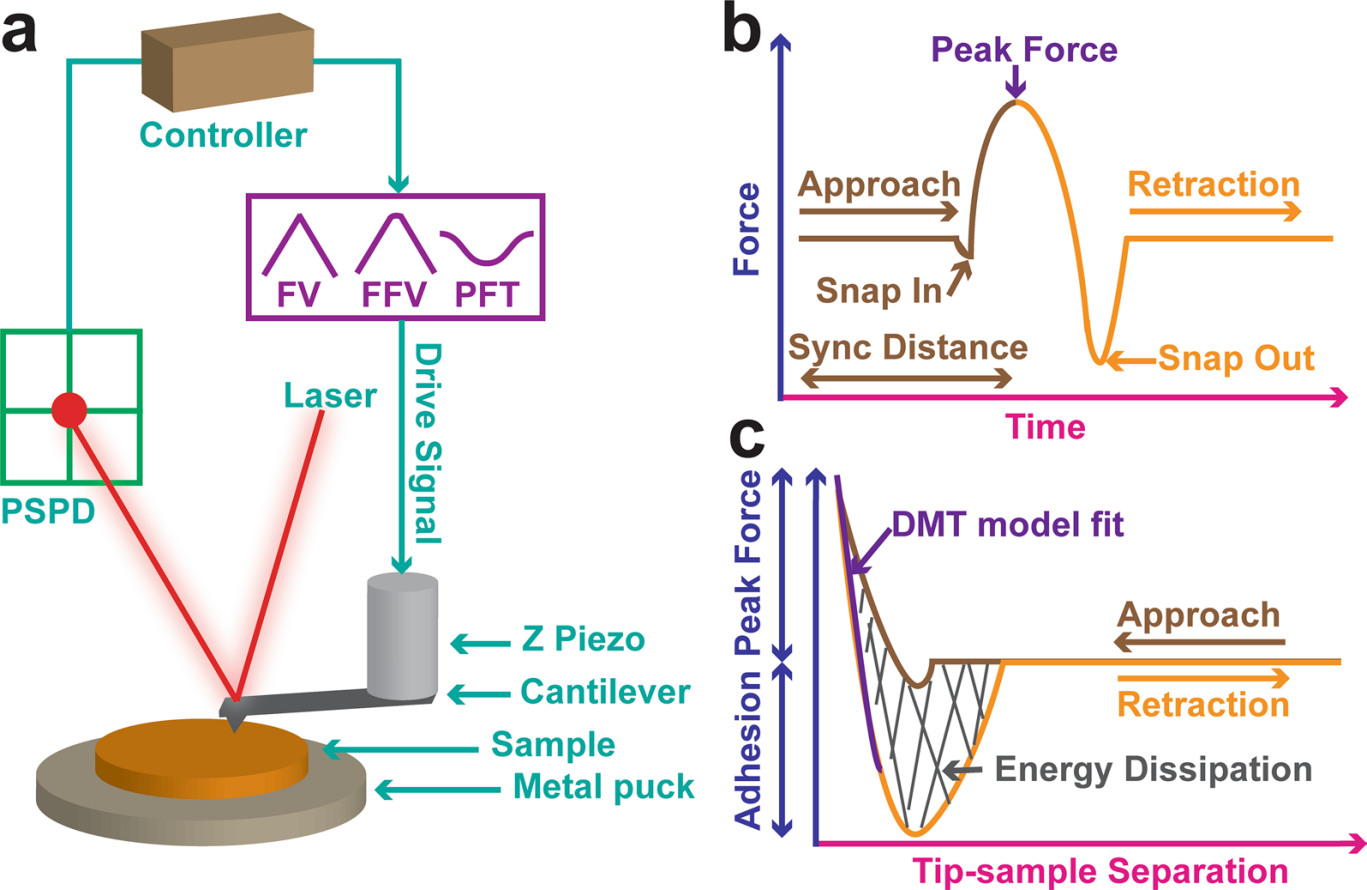
Mechanism of AFM force ramp, experimental design, data acquisition and interpretation. The LDPE sample was mounted onto a metal puck. A sharp cantilever tip indented into the LDPE sample and retracted away when a preset applied force was reached (**a**). A laser shined from the top, hit and deflected off from the back side of the cantilever. The deflection signal was received by a position sensitive photodiode (PSPD). The cantilever motion was driven by the attached Z piezo. Depending on the choice of technique, the drive signal could be a triangular wave (FV), a corner-rounded triangular wave (FFV) or a sine wave (PFQNM). The PFQNM force measurement was schematically depicted in **b**, Force versus time plot clearly illustrated the tip underwent a snap-in contact when brought close the sample surface and a snap-out of contact when retracted away from the sample surface. The Sync Distance was a time constant at which the height sensor reached the lowest position. The force versus Z displacement curve (F-Z) was recorded by the AFM and further converted into the force versus tip-sample separation (F-D) curve (**c**). The DMT modulus was extracted by fitting the contact part of retraction curve with DMT model. The integration over the hysteresis loop was referred to as energy dissipation

To quantitatively map out the mechanical properties of the LDPE sample (Fig. 1), the experiment was designed in such a way that a sharp cantilever tip indented into the LDPE sample and withdrew away from the sample surface when a preset force load was achieved (Fig. 1a). The force was recorded by detecting the vertical deflection signal in the position sensitive photodiode (PSPD). The cantilever motion was driven by the Z piezo movement. Depending on the choice of technique, the drive signal could be a triangular wave (FV), a corner-rounded triangular wave (FFV) or a sine wave signal (PFQNM). The PFQNM was schematically drawn in Fig. 1b, force versus time curve clearly demonstrated the tip underwent a snap-in contact when approaching the sample surface and a snap-out of contact when retracting away from the sample surface. The Sync Distance defined the turning point that separated the approach curve from the retraction curve. On a hard surface, this point was a time constant when the Z piezo reached the lowest position. It also meant when the force reached the peak force. In contrast, on a soft compliant sample this point could shift a little due to time dependent sample deformation. Regardless of the techniques adopted, AFM recorded the force versus Z displacement curve and was further converted into the force versus tip-sample separation curve (Fig. 1c). The contact part of the retraction curve was fitted with a linearized DMT model described below and the DMT modulus was extracted. The energy dissipation was calculated by integrating the hysteresis loop. A cantilever with a proper spring constant was chosen prudently so that the cantilever tip is able to indent into the sample yet has enough force sensitivity. On the other hand, tip radius needs to be considered as well because the applied stress is also dependent on contact area. In light of these, RTESPA-150 probes were selected because it produces right amount of force to indent into the sample but preserve high force sensitivity at the same time.

### Data Analysis

Offline data analysis was conducted with the NanoScope Analysis software (Bruker Co., Santa Barbara, CA) supplied by the AFM factory. All topographical images were subject to first order flatten that gets rid of Z piezo drift, background noise as well as corrects sample tilt. The surface roughness was evaluated by surface roughness feature provided by the NanoScope Analysis software.

2 $$R_{{\text{q}}} = \sqrt {\frac{{\sum \left( {Z_{{\text{i}}} - Z_{{\text{m}}} } \right)^{2} }}{N}}$$

where $N$ is the total number of points within the image area, $Z_{{\text{i}}}$ is the $Z$ height of the *i*th data point, and $Z_{{\text{m}}}$ is the mean $Z$ height over the whole area. All mechanical data images were left intact without levelling.

Both force ramp, fast force volume and PFQNM yielded force versus Z piezo displacement (F-Z) curves. Force versus tip-sample separation (F-D) curves are more physically meaningful and demanded for model fit. The Z displacement consists of three components, namely tip-sample separation (*D*), cantilever deflection (*d*), and indentation depth ($\delta$). The conversion of F-Z to F-D requires subtracting cantilever deflection (*d*), and indentation depth ($\delta$) from Z displacement. It can be done either in real time control software or by offline data analysis software, providing the cantilever deflection sensitivity and spring constant have been calibrated. In addition, baseline correction function was executed to offset the force curve baseline to zero. Finally, F-D curves were obtained and subject to DMT model fit. According to the Hertzian contact theory,

3 $$F_{{{\text{appl}}}} = \frac{4}{3}E_{r} \sqrt R \delta ^{{\frac{3}{2}}} + F_{{{\text{adh}}}}$$

where $F_{{{\text{appl}}}}$ is the force that tip applied on the sample. Adhesion force ($F_{{{\text{adh}}}}$) is taken into account. $R$ is the tip radius for the presumed sphere tip. $\delta$ is the indentation depth. $E_{{\text{r}}}$ is the reduced Young’s modulus. It is related to tip’s and sample’s moduli,

4 $$\frac{1}{{E_{{\text{r}}} }} = \left( {\frac{{1 - v_{{\text{s}}}^{2} }}{{E_{{\text{s}}} }}} \right) + \left( {\frac{{1 - v_{{\text{t}}}^{2} }}{{E_{{\text{t}}} }}} \right)$$

where $v_{{\text{s}}}$ and $v_{{\text{t}}}$ are the Poisson’s ratios of the sample and the AFM tip respectively. $E_{{\text{s}}}$ and $E_{{\text{t}}}$ are the Young’s moduli of sample and AFM tip, respectively. The tip’s Young’s modulus is several orders of magnitude larger than that of the LDPE sample, so the tip term can be neglected. Once $E_{{\text{r}}}$ and $v_{{\text{s}}}$ are known, $E_{{\text{s}}}$ can be readily calculated.

By taking both sides of Eq. (3) to the $\frac{2}{3}$ power after subtracting the $F_{{{\text{adh}}}}$ from $F_{{{\text{appl}}}}$, a linearized model has been employed to fit all force data [30]. This model does not require identification of contact point.

5 $$\left( {F_{{{\text{appl}}}} - F_{{{\text{adh}}}} } \right)^{{\frac{2}{3}}} = \left( {\frac{4}{3}E_{{\text{r}}} \sqrt R } \right)^{{\frac{2}{3}}} \delta$$

Then $E_{{\text{r}}}$ and $E_{{\text{s}}}$ were extracted as a result.

6 $$E_{{\text{r}}} = \frac{3}{4}\left( {\frac{{\left( {F_{{{\text{appl}}}} - F_{{{\text{adh}}}} } \right)^{{\frac{2}{3}}} }}{\delta }} \right)^{{\frac{3}{2}}} \frac{1}{{\sqrt R }} = \frac{3}{4}\;{\text{slope}}^{{\frac{3}{2}}} \frac{1}{{\sqrt R }}$$

The applied force was calculated from Hooke’s law since cantilever acted like a spring.

7 $$F_{{{\text{appl}}}} = k \times d$$

where $k$ is the cantilever spring constant and $d$ is the cantilever deflection, which was calculated by multiplying cantilever deflection sensitivity with vertical deflection signal.

For creep compliance analysis, the Voigt version of the SLS model was adopted [19]. In this three-element model, a spring (*E*_1_) is in series with a spring (*E*_2_)-dashpot Voigt element in parallel. The compression distance (*d*) as a function of time can be described as:

8 $$d(t) = \frac{F}{{k_{1} }} + \frac{F}{{k_{2} }} \times \left( {1 - {\text{e}}^{{ - \frac{{tk_{2} }}{\eta }}} } \right)$$

where *F* is the total loading force, *k*_1_ and *k*_2_ are the elasticity of *E*_1_ and *E*_2_, respectively. *η* represents the viscosity of the dashpot. Since the tip-sample interaction area is a finite area, not a single point. The model can be improved by rewriting the equation in terms of stress, strain and modulus. The method developed by Lam and colleagues was adopted in this study. Their analogous equation is:

9 $$\varepsilon (t) = \frac{\sigma }{{E_{1} }} + \frac{\sigma }{{E_{2} }} \times \left( {1 - {\text{e}}^{{ - \frac{{tE_{2} }}{\eta }}} } \right)$$

where *ε*(*t*) denotes strain as a function time, *σ* is stress. *E*_1_ and *E*_2_ are the instantaneous and delayed elastic moduli, respectively. *η* represents the viscosity of the dashpot. Moreover, stress *σ* and strain *ε* are related with modulus *E* or compliance *D* by the following relationship.

10 $$E = \frac{\sigma }{\varepsilon } = \frac{1}{D}$$

Equation (9) can therefore be rewritten as:

11 $$D = \frac{1}{E} = \frac{1}{{E_{1} }} + \frac{1}{{E_{2} }} \times \left( {1 - {\text{e}}^{{ - \frac{{tE_{2} }}{\eta }}} } \right)$$

where *D* and *E* denote the creep compliance and the combined elastic modulus of the system, respectively. Rewrite Eq. (5) as

12 $$\delta = \left( {\frac{{3\left( {F_{{{\text{appl}}}} - F_{{{\text{adh}}}} } \right)}}{{4\sqrt R E_{{\text{r}}} }}} \right)^{{\frac{2}{3}}}$$

Substituting Eq. (11) into Eq. (12) gives rise to

13 $$\delta \left( t \right) = \left\{ {\frac{{3\left( {F_{{{\text{appl}}}} - F_{{{\text{adh}}}} } \right)}}{{4\sqrt R }} \times \left( {\frac{1}{{E_{1} }} + \frac{1}{{E_{2} }} \times \left( {1 - {\text{e}}^{{ - \frac{{tE_{2} }}{\eta }}} } \right)} \right)} \right\}^{{\frac{2}{3}}}$$

The creep data can be fitted with Eq. (13) and the retardation time *τ* can be derived using

14 $$\tau = \frac{\eta }{{E_{2} }}$$

The retardation time is referred to as the time at which ~ 63% of creep has occurred.

All force measurements were repeated three times. Results were reported in the form of Mean ± SD (standard deviation) while number of independent experiments was denoted as *n* = 3.

## Results

**Fig. 2:**
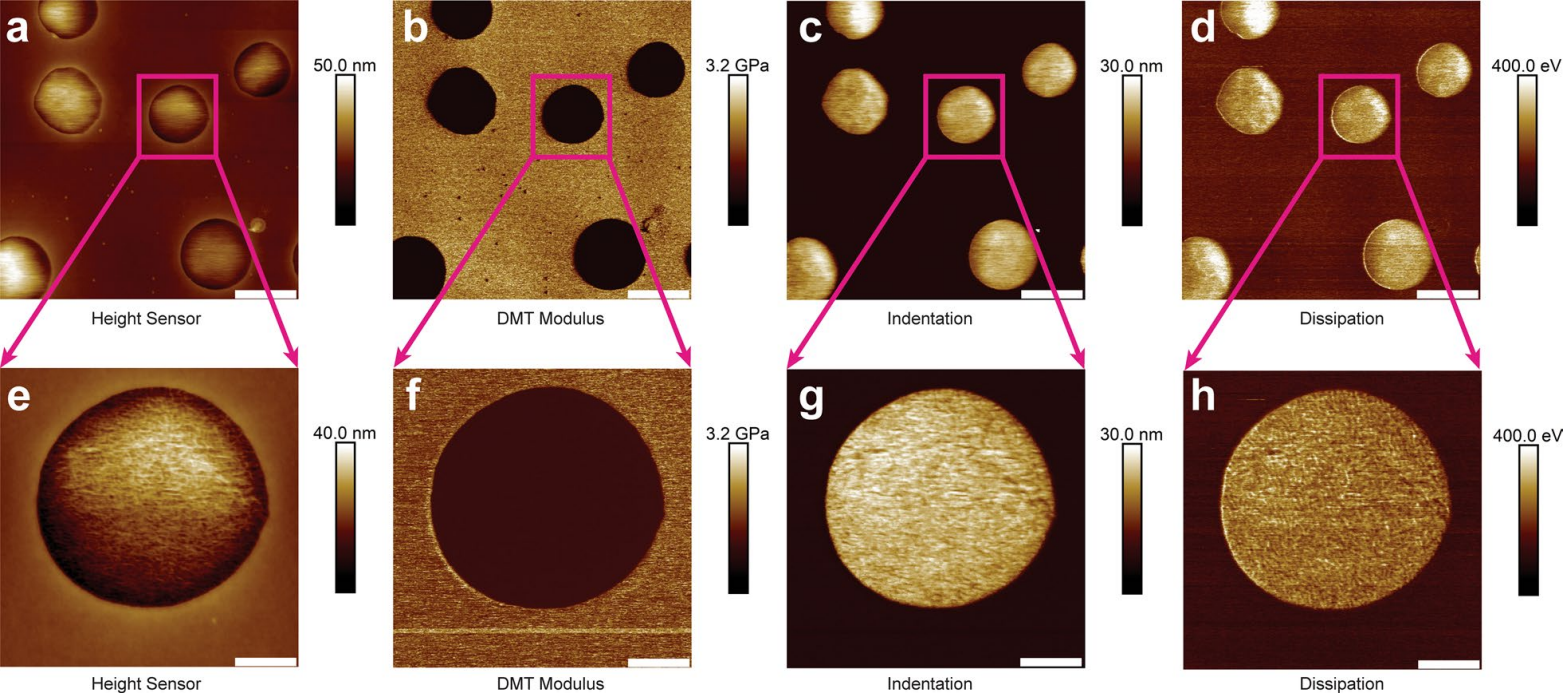
Representative PFQNM nanomechanical mapping (5 µm × 5 µm) of PS/LDPE blend sample at 2 kHz. Panels **a**–**d** are height sensor image, DMT modulus channel, indentation channel, and energy dissipation channel. For images **a**–**d**, the scale bars represent 1 µm. On the completion of the survey scan, the AFM is guided to physically zoom in on the LDPE region and take a high-resolution small size (1.3 µm × 1.3 µm) scan. The corresponding image channels are displayed in panels **e–h**. The scale bar represents 260 nm for panels **e**–**h**

To evaluate the effectiveness and accuracy of PFQNM, a large survey scan with 5 µm × 5 µm was performed. Representative PFQNM images of PS/LDPE blend sample at 2 kHz were assembled in Fig. 2. Figure 2a–d were height sensor image, DMT modulus channel, indentation channel, and energy dissipation channel. The flat region was the PS component while the bulging region was the LDPE (Fig. 2a). On the completion of the survey scan, the AFM was instructed to physically zoom in on the LDPE region and take a high-resolution small size (1.3 µm × 1.3 µm) scan. The corresponding image channels were displayed in Fig. 2e–h.

**Fig. 3:**
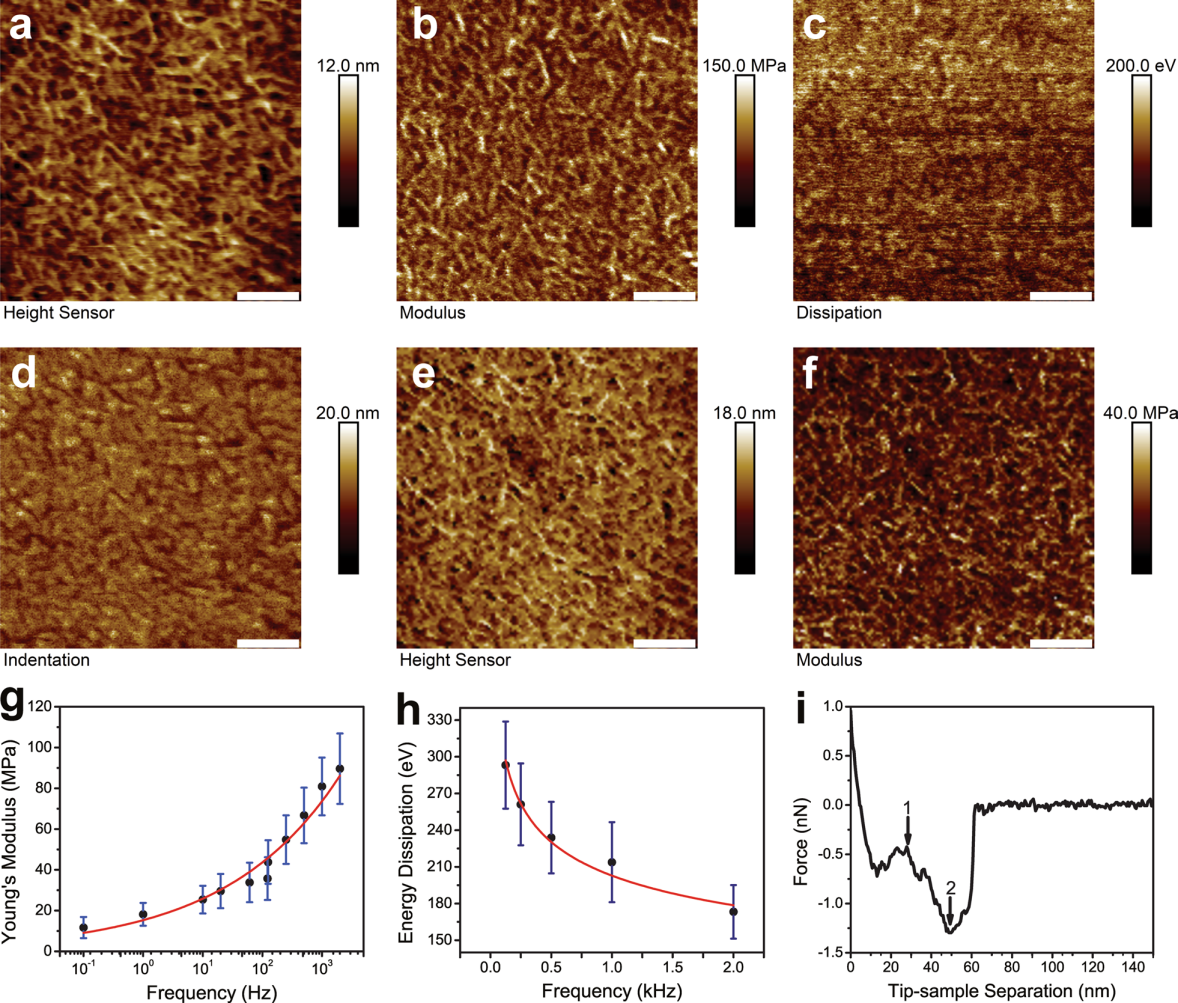
Mechanical property of LDPE sample mapped at different frequencies. Panels **a**–**d** were height sensor image, DMT modulus channel, energy dissipation, and indentation channel captured with PFQNM at 2 kHz on neat LDPE region. Surface roughness of the height sensor image was reported in the form of $R_{{\text{q}}}$ as 2.58 ± 0.35 nm. Panels e and f were height sensor image and DMT modulus channel captured with FFV at 122 Hz on neat LDPE region. For images **a**–**f**, the scale bars represented 100 nm. Relationship between measured Young’s modulus (*E*) and the force mapping frequency (*f*) was plotted in **g**. The measured Young’s moduli at different frequencies were tabulated in Table 1. The data were fitted with a power function yielded $E = 15.31 \times f^{{0.23}}$ ($R^{2}$ = 0.96). The relationship between energy dissipation (*E*_diss_) and different mapping frequencies (*f*) was shown in panel **h**. The energy dissipation values obtained at 2 kHz, 1 kHz, 0.5 kHz, 0.25 kHz and 0.125 kHz were 173.2 ± 21.9 eV, 213.8 ± 32.7 eV, 233.9 ± 29.3 eV, 261.1 ± 33.5 eV, 293.2 ± 35.6 eV, respectively. The data were fitted with a power function yielded $E_{{{\text{diss}}}} = 202.83 \times f^{{ - 0.18}} ~$ ($R^{2}$ = 0.97). A representative F-D curve showed two distinct ruptures of AFM tip from LDPE sample surface (panel **i**). The occurrence of multiple ruptures took place more frequently at lower frequencies, i.e. 0.1–1 Hz

Next, PFQNM, FV and FFV were conducted over a neat LDPE region at 0.5 µm × 0.5 µm. A representative set of PFQNM at 2 kHz were collected in Fig. 3a–d. They included height sensor, modulus mapping, energy dissipation, and indentation. Surface roughness of the height sensor image was reported in the form of $R_{{\text{q}}}$ as 2.58 ± 0.35 nm. Another representative set of FFV at 122 Hz were shown in Fig. 3e, f. Note there were no energy dissipation and indentation channels for FV and FFV. The elastic moduli at different frequencies were pooled together (Fig. 3g). Young’s moduli data were reported in Table 1. The Young’s moduli at 0.1 Hz, 1 Hz, 10 Hz, 20 Hz, 61 Hz, 122 Hz, 125 Hz, 250 Hz, 500 Hz, 1 k Hz and 2 k Hz were 11.7 ± 5.2 MPa (*n* = 3), 18.2 ± 5.6 MPa (*n* = 3), 25.4 ± 6.8 MPa (*n* = 3), 29.6 ± 8.4 MPa (*n* = 3), 33.8 ± 9.7 MPa (*n* = 3), 35.7 ± 10.5 MPa (*n* = 3), 43.8 ± 10.7 MPa (*n* = 3), 54.8 ± 11.9 MPa (*n* = 3), 66.7 ± 13.6 MPa (*n* = 3), 80.9 ± 14.2 MPa (*n* = 3), 89.6 ± 17.3 MPa (*n* = 3), respectively. The scatter plot was generated with Origin 8.5 software. The data were fitted with a power function yielded $E = 15.31 \times f^{{0.23}}$ ($R^{2}$ = 0.96). The relationship between energy dissipation and different mapping frequencies was plotted in Fig. 3h. The energy dissipation values obtained at 2 kHz, 1 kHz, 0.5 kHz, 0.25 kHz, and 0.125 kHz were 173.2 ± 21.9 eV (*n* = 3), 213.8 ± 32.7 eV (*n* = 3), 233.9 ± 29.3 eV (*n* = 3), 261.1 ± 33.5 eV (*n* = 3), 293.2 ± 35.6 eV (*n* = 3), respectively. The data were fitted with a power function yielded $E_{{{\text{diss}}}} = 202.83 \times f^{{ - 0.18}} ~$ ($R^{2}$ = 0.97). A representative F-D curve showed two distinct ruptures of AFM tip from LDPE sample surface (Fig. 3i). The occurrence of multiple ruptures took place more frequently at lower frequencies, i.e. 0.1–1 Hz.

**Table 1:** Measured Young’s modulus at different frequencies (Mean ± SD, *n* = 3)

Frequency (Hz)	Modulus (MPa)
0.1	11.7 ± 5.2
1	18.2 ± 5.6
10	25.4 ± 6.8
20	29.6 ± 8.4
61	33.8 ± 9.7
122	35.7 ± 10.5
125	43.8 ± 10.7
250	54.8 ± 11.9
500	66.7 ± 13.6
1000	80.9 ± 14.2
2000	89.6 ± 17.3

**Fig. 4:**
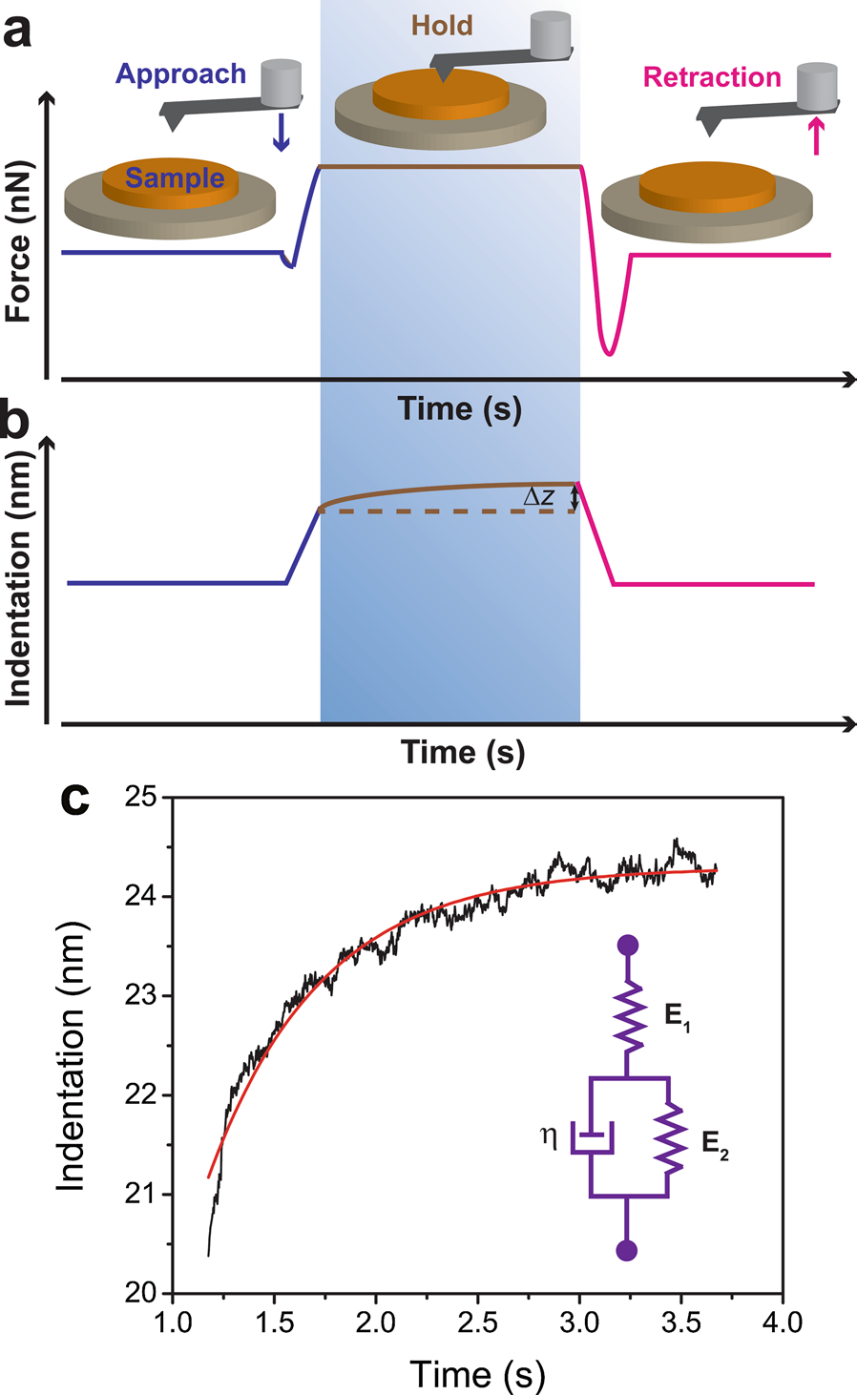
Creep compliance measurement on a neat LDPE region of the PS/LDPE sample. The working principle of AFM creep experiment was illustrated in panel **a**. Initially, the AFM tip was brought into contact with sample surface till it reached the predefined force setpoint. The tip was then held onto the sample for a certain time period, during which the force was kept constant. Following that, the tip was retracted. In the hold segment, the AFM recorded the change in Z motion (panel **b**). The change in indentation depth as a function of time could be fitted with Voigt version of SLS model using Eq. (13). A representative creep curve was shown in panel **c**. The black curve was the data, while the red solid line was the fitting curve. The inset indicated the Voigt version of SLS model, featuring a spring (*E*_1_) in series with a spring (*E*_2_)-dashpot (*η*) Voigt element in parallel

Lastly, creep compliance measurement was carried on a neat LDPE region of the PS/LDPE sample. The working principle of AFM creep experiment was illustrated in Fig. 4a. Initially, the AFM tip was brought into contact with sample surface until the predefined force setpoint was reached. The tip was sthen held onto the sample for a certain time period, during which the force was kept constant. Following that, the tip was retracted. In the hold segment, the AFM recorded the change in Z motion. The change in indentation depth as a function of time (Fig. 4b) could be fitted with Voigt version of SLS model using Eq. (13). A representative creep curve was shown in Fig. 4c. The black curve was the data while the red solid line was the fitting curve. The inset indicated the Voigt version of SLS model, featuring a spring (*E*_1_) in series with a spring (*E*_*2*_)-dashpot (*η*) Voigt element in parallel. The experiment showed that instantaneous elastic modulus *E*_1_, delayed elastic modulus *E*_2_, viscosity *η*, retardation time *τ* were 22.3 ± 3.5 MPa, 43.3 ± 4.8 MPa, 38.7 ± 5.6 MPa‧s and 0.89 ± 0.22 s, respectively. The data were tabulated in Table 2.

**Table 2:** Viscoelastic parameters measured by creep experiment (Mean ± SD, *n* = 3)

Instantaneous elastic modulus *E*_1_ (MPa)	Delayed elastic modulus *E*_2_ (MPa)	Viscosity *η *(MPa s)	Retardation time *τ* (s)
22.3 ± 3.5	43.3 ± 4.8	38.7 ± 5.6	0.89 ± 0.22

## Discussion

In the present study, a comprehensive powerful nanomechanical mapping approach for polymer samples has been developed by incorporating a number of nanoscale AFM based force measurements. The approach allows simultaneous high-resolution topography imaging and quantitative nanomechanical mapping. Local mechanical behavior can be correlated with sample topography. More importantly, the time dependent mechanical response of soft viscoelastic materials has been successfully mapped out. The Hertz model is a widely received contact mechanics model [31], in which the scenario when a rigid probe indents a semi-infinite, isotropic, homogeneous elastic surface is described. However, the Hertz model assumes no surface forces, which is not true for soft materials. To overcome this shortcoming, the Johnson–Kendall–Roberts (JKR) model and the DMT model have been developed. Given the setup in this study, the DMT model can be implemented as there are high elastic modulus, low adhesion, and small tip radius involved where long rang surface forces exist. The force setpoint at 5 nN has been empirically obtained, and justified to be the optimum value in terms of getting meaningful indentation depth while the DMT model still holds. Low force load also gives rise to sample deformation in elastic regime not plastic regime. In addition, sharp tip enables high resolution sample topography imaging in PFQNM measurements, which is an attractive advantage when correlates sample topography with mechanical properties.

Tip radius estimation is not trivial in quantitative mechanical measurements. Many researches estimate the tip radius by backward calculation using a sample with known modulus [29, 32]. This work adopts a different reconstruction strategy that does not require such a sample. It has been documented that using blunt tips tend to yield tighter modulus numbers and that sharp tips may overestimate the modulus. However, sharp tips preserve high spatial resolution, an advantage not possessed by other techniques. Polymer fibrils are clearly seen (see a 0.5 µm × 0.5 µm scan in Fig. 3). Sharp tips, even under small load, can penetrate into compliant samples due to large stress, resulting in large indentation. Therefore, it could compromise the validity of the DMT model. That is not the case in this study as the applied force is controlled in a precise and sensitive manner, evidenced by the resulted indentation depth and the effective tip radius in the same order of magnitude (22.5 ± 3.2 nm, *n* = 3). Surface roughness ($R_{{\text{q}}}$) of the LDPE height image is 2.58 ± 0.35 nm, indicating the surface is flat and surface roughness should not be treated as a confounding factor to quantitative measurements [33]. In addition, the linearized DMT model fit does not require determination of the contact point that could otherwise lead to major errors in the final calculated modulus [34]. Taken together, the current experiment setup fulfills the DMT model.

To evaluate the effectiveness of PFQNM, the PS/LDPE sample has been scanned at large size. The survey scan shows LDPE has higher adhesion than PS (Fig. 2b), suggesting LDPE is stickier. AFM tip indents deeper in LDPE than in PS (Fig. 2c), indicating LDPE is softer than PE. The determined Young’s moduli for LDPE and PS are about 90 MPa and 2.5 GPa, respectively. The PS region is a little stiff for RTESPA-150 probe to indent, thus the measured modulus tends to be higher than the nominal value. Both PFQNM and FFV generate high resolution topography and modulus images (c.f. Fig. 3a, b, e, f). It is noteworthy that FFV requires reasonable data acquisition time, although it is not as impressive as PFQNM but much faster than traditional force ramp. Energy dissipation is an observable that explicitly demonstrates how much energy loss per tapping cycle (Fig. 3h). The more viscoelastic of the material, the more energy loss it incurs. The energy dissipation map demonstrates that AFM probe loses more energy on LDPE than on PS, implying LDPE is viscoelastic and response time plays an important role. The relaxation function for the power-law rheology model is described as $\varphi = E_{{\text{a}}} \left( {\frac{t}{{t_{0} }}} \right)^{{ - \gamma }}$ [35], where *E*_a_ is the apparent Young’s modulus at time *t*_0_, is the power-law exponent *γ* and *t*_0_ is a timescale factor which is set to 1 s. The dimensionless number *γ* characterizes the viscoelastic behavior of the material, with *γ* = 0 for purely elastic solid and *γ* = 1 for purely Newtonian fluid [36]. Current study indicates LDPE has more elastic behavior than viscous counterpart. Figure 3i exhibits an interesting finding in FV experiments that a force curve harboring two rupture events. The multiple rupture events occur more frequently in lower frequencies, i.e. 0.1–1 Hz. It is conceivable that with lower frequency, the tip dwells longer on sample surface that results in forming stronger bonds. When tip is retracted, the slower motion of tip would break the bonds at lower speed, providing the chance of being captured by AFM [37]. On the contrary, when performed at higher frequencies, weaker bonds are formed due to short dwell period and AFM is not capable of capturing transition rupture events due to poor temporal resolution. Another plausible explanation is that the combination of force exerted and longer interaction time on sample induces polymer chain conformation change, as reported previously that force induces rotation of carbon–carbon double bonds [38]. With piconewton force sensitivity and sub-nanometer distance accuracy, F-D curves not only reveal the strength of the formed bonds but also shed insights into the elastic properties and conformational changes. It was documented that at low forces (< 100 pN) and large forces (> 300 pN) the mechanical behavior of polymer chains is majorly affected by its entropic elasticity and enthalpic elasticity, respectively [39].

To further investigate the time dependent mechanical response of LDPE, creep compliance experiment has been carried out on the premise that the closed-loop scanner has been drift calibrated. Experimental data show that instantaneous elastic modulus *E*_1_, delayed elastic modulus *E*_2_, viscosity *η*, retardation time *τ* are 22.3 ± 3.5 MPa, 43.3 ± 4.8 MPa, 38.7 ± 5.6 MPa s and 0.89 ± 0.22 s, respectively (Table 2). This set of values for creep behavior is close to those reported for polyurethane nanocomposites [40] and syndiotactic polypropylene [41] and higher than those for bacterial biofilm [19] and live cells [36, 42]. While large AFM indenter platform measures elastic modulus of soft samples in an ensemble way, it does not enjoy high spatial resolution of elasticity. Such local mechanical properties are critical for some specimen. For instance, cell membranes are composed of various substructures like cytoskeleton, filament network and microvilli, each has varying elasticities [30]. A recent paper has studied the elastic modulus of fibroblast cells in the frequency range of 0.3–250 Hz [43]. The authors have discovered raised apparent Young’s modulus when ramp frequency increased, consistent with the observations of current study. The approaches reported here are as reliable as any other nanomechanical techniques provided the force-indentation has been prudently designed and the data analysis has been carefully executed. The PFQNM measurement is particularly helpful due to its localized correlation of sample topography with mechanical behavior. It is advantageous in terms of local non-destructive probing of mechanical properties over traditional instrumented indentation, where large probe tip is used and large destructive force is applied. Furthermore, the AFM creep experiment provides dynamic mechanical behavior at nanoscale. The methodology presented here offers multiparametric, multifunctional probing of mechanical measurement along with exceptional high spatial resolution. It has been successfully exploited for quantitative nanomechanical mapping of soft polymers such as LDPE, and can potentially be extended to complex biological systems [43–45].

## Conclusions

Utilizing state-of-the-art PFQNM as well as with FV and FFV, the power-low rheology of a LDPE sample has been evaluated in a time-dependent fashion. Specifically, rigorous calibrations are done. Force data are fitted with a linearized DMT contact mechanics model considering surface adhesion force. Elastic Young’s modulus was measured at frequencies spanned four orders of magnitude. Increased Young’s modulus was discovered with increasing acquisition frequency. The Young’s modulus is 11.7 ± 5.2 MPa at 0.1 Hz but increases to 89.6 ± 17.3 MPa at 2 kHz. The acquisition frequency dependent modulus change could be described by a power function $E = 15.31 \times f^{{0.23}}$ ($R^{2}$ = 0.96). Energy dissipation in the range of 0.125–2 kHz further supports this observation. Furthermore, creep compliance experiment shows that instantaneous elastic modulus *E*_1_, delayed elastic modulus *E*_2_, viscosity *η*, retardation time *τ* are 22.3 ± 3.5 MPa, 43.3 ± 4.8 MPa, 38.7 ± 5.6 MPa‧s and 0.89 ± 0.22 s, respectively. The multiparametric, multifunctional local probing of mechanical measurement along with exceptional high spatial resolution imaging open new opportunities for quantitative nanomechanical mapping of soft polymers, and can potentially be extended to biological systems.

## Data Availability

The datasets used or analyzed during the current study are available from the corresponding author on reasonable request.

## Abbreviations

AFM: Atomic force microscopy
DMT: Derjaguin–Muller–Toporov
FFV: Fast force volume
FV: Force volume
JKR: Johnson–Kendall–Roberts
LDPE: Low density polyolefin
PFQNM: PeakForce quantitative nanomechanical mapping
PFT: PeakForce tapping
PS: Polystyrene
